# Supplementary material for: Open datasets and code for multi-scale relations on structure, function and neuro-genetics in the human brain
Source: Sci Data. 2024 Feb 29;11:256. doi: 10.1038/s41597-024-03060-2 (PMC10904384; doi:10.1038/s41597-024-03060-2)
Supplement: Supplementary file 1 — Supplementary figures [file 41597_2024_3060_MOESM1_ESM.pdf]

Supplementary Figures

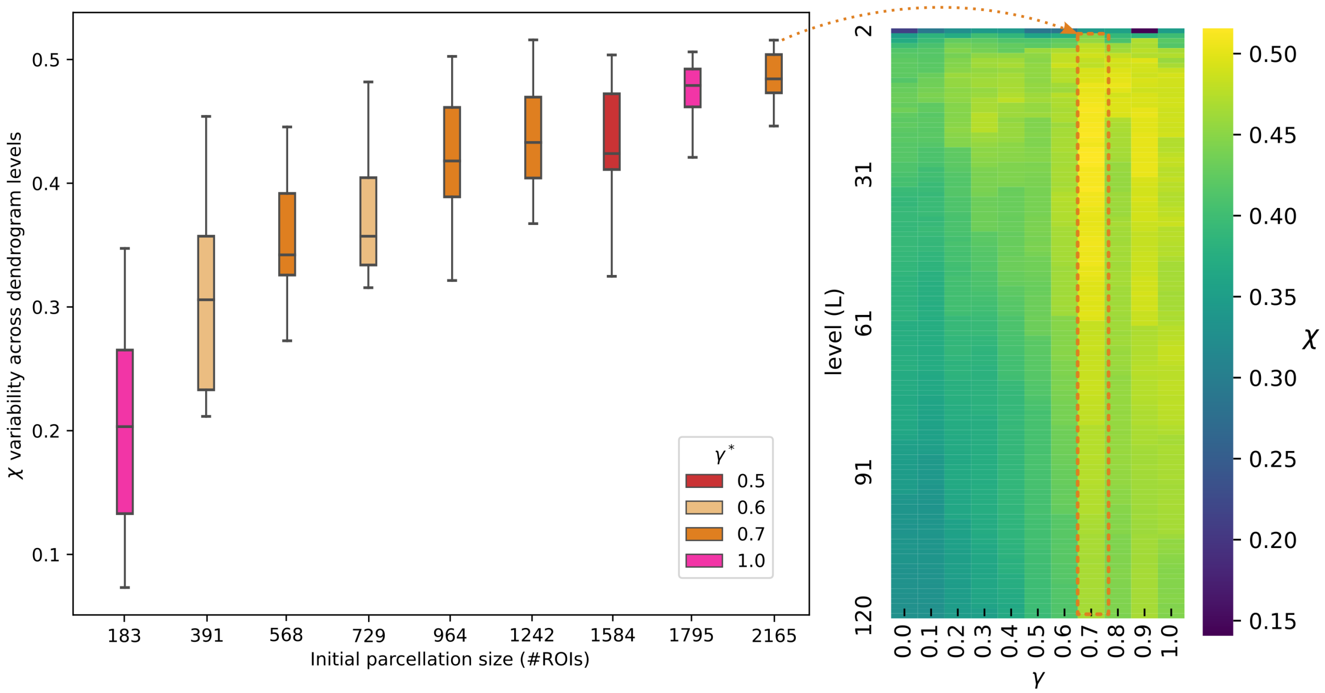

Figure S1. Selection of best initial parcellation atlas and optimal value of γ parameter. **Left:** Box-plots of the cross-modularity χ across different dendrogram levels and for different size of the initial parcellation atlas (iPA). The different values of χ, on which the box-plots are calculated, come from the different levels in the dendrogram (here we have varied from 2 to 120). As the number of ROIs for the initial parcellation increases, the cross-modularity χ also increases. **Right:** The optimal value of $\gamma^* = 0.7$ is also illustrated for the same range of dendrogram levels, ie. from 2 to 120.

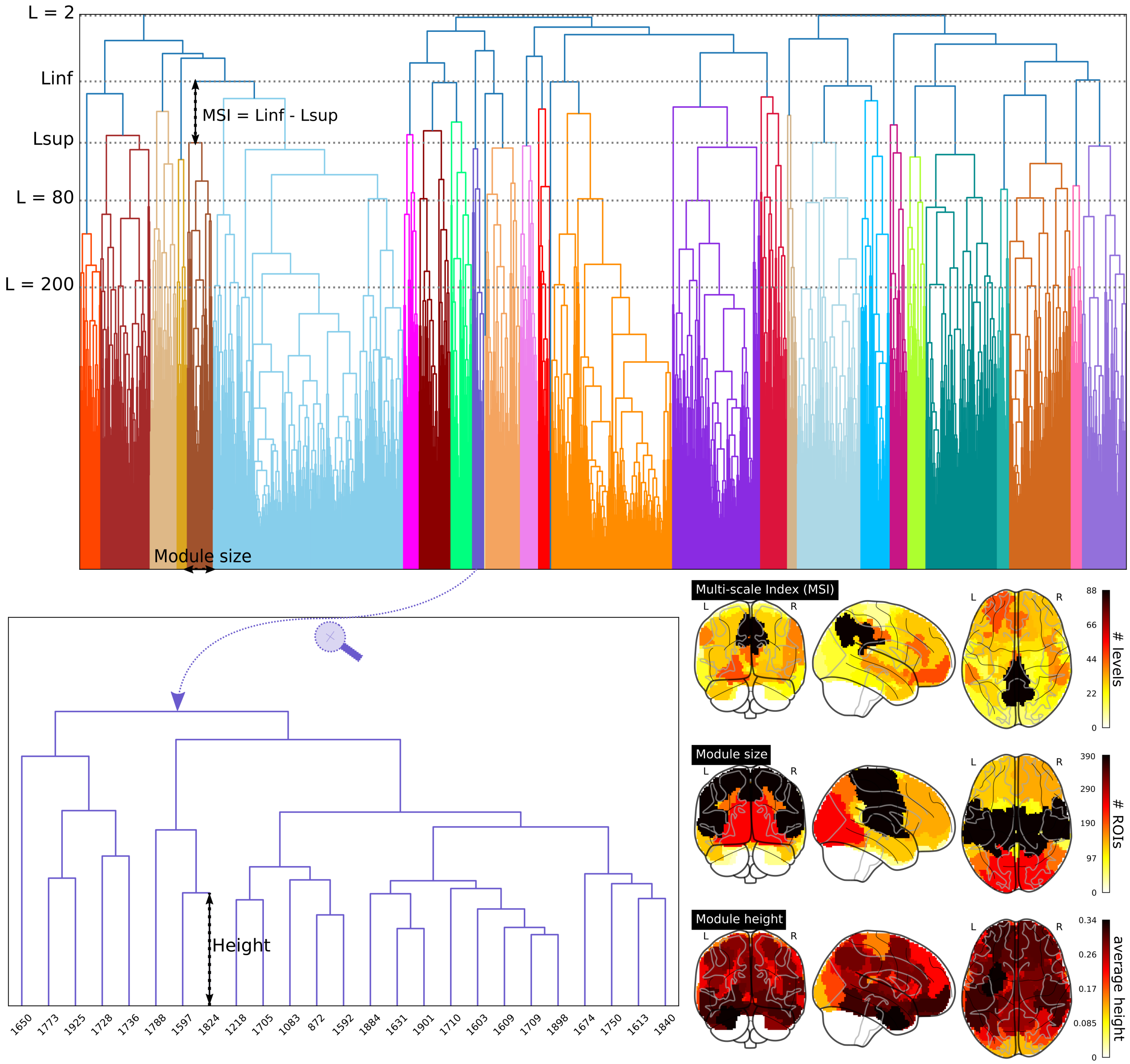

Figure S2. Dendrogram measures and module analysis in the the optimal brain parcellation. Each color in the dendrogram corresponds to a distinct module (that for the optimal brain partitions correspond to 26 different modules). The y-axis of the dendrogram illustrates different levels (L) and provides visual representation of a given module and their inferior (Linf) and superior (Lsup) levels. Different multi-scale metrics can be defined, eg., higher values of the Multi-scale Index (MSI) correspond to enhanced preservation of the module throughout multiple levels in the dendrogram, indicating heightened stability across the tree structure. The module size is also represented by the width of each module in the dendrogram. The lower inset shows a marked micro-region Height indicating to which extent a given micro-region is detached from the overall tree structure. Lower values of Height represent stronger connectivity of the micro-region with the other micro-regions belonging to the same module. The figure also shows brain maps depicting the three aforementioned measures for each of the modules.

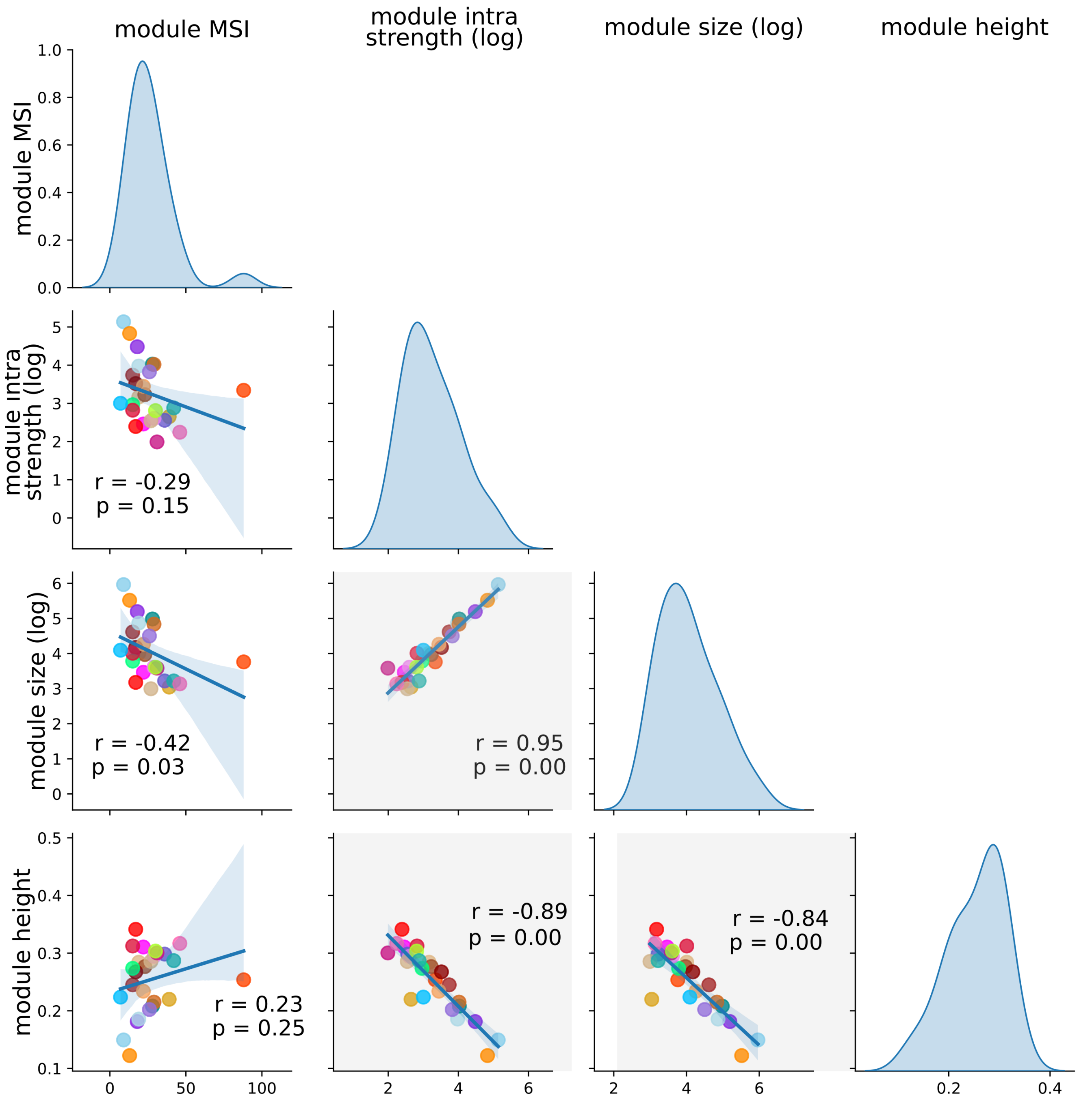

Figure S3. Statistical dependencies between different multi-scale metrics. Cross-correlation plots between multi-scale index (MSI), intra-strength (as a proxy for module segregation), size and height. Different color-points represent different modules of the optimal brain parcellation with a total number of 26 modules. Principal-diagonal plots show probability density functions of the different metrics. Notice that higher correlations between module size, module height, and intra-strength indicate that heightened connectivity within modules (segregation) results in larger dendrogram modules and delayed micro-region splitting in the tree. The MSI reveals a distinct outlier (M1) encompassing several structures such as a part of the precuneus, isthmus cingulate, and posterior cingulate, located at the border between DMN, fronto-parietal, and dorsal attention networks.
